# Domain-Specific Control of Selective Attention

**DOI:** 10.1371/journal.pone.0098260

**Published:** 2014-05-27

**Authors:** Szu-Hung Lin, Yei-Yu Yeh

**Affiliations:** Department of Psychology, National Taiwan University, Taipei City, Taiwan; Centre de Neuroscience Cognitive, France

## Abstract

Previous research has shown that loading information on working memory affects selective attention. However, whether the load effect on selective attention is domain-general or domain-specific remains unresolved. The domain-general effect refers to the findings that load in one content (e.g. phonological) domain in working memory influences processing in another content (e.g., visuospatial) domain. Attentional control supervises selection regardless of information domain. The domain-specific effect refers to the constraint of influence only when maintenance and processing operate in the same domain. Selective attention operates in a specific content domain. This study is designed to resolve this controversy. Across three experiments, we manipulated the type of representation maintained in working memory and the type of representation upon which the participants must exert control to resolve conflict and select a target into the focus of attention. In Experiments 1a and 1b, participants maintained digits and nonverbalized objects, respectively, in working memory while selecting a target in a letter array. In Experiment 2, we presented auditory digits with a letter flanker task to exclude the involvement of resource competition within the same input modality. In Experiments 3a and 3b, we replaced the letter flanker task with an object flanker task while manipulating the memory load on object and digit representation, respectively. The results consistently showed that memory load modulated distractibility only when the stimuli of the two tasks were represented in the same domain. The magnitude of distractor interference was larger under high load than under low load, reflecting a lower efficacy of information prioritization. When the stimuli of the two tasks were represented in different domains, memory load did not modulate distractibility. Control of processing priority in selective attention demands domain-specific resources.

## Introduction

Selective attention is crucial in everyday activities. While driving, people need to focus on the signs, the pedestrians, the distance to the car in front, whether there is enough space for changing lanes, and other relevant information to drive safely. The ability to control the priority of information processing without interference from task-irrelevant stimuli is especially important for higher cognitive functions, such as learning, reasoning, and decision making. As many would testify, the ability to do so may be difficult when the mind is occupied by something else. Maintaining information in working memory may cause difficulty in selective attention. Empirical evidence supports this daily observation, with lower efficiency in selective attention when more information is maintained in working memory under high load than when less information is maintained under low load [1], [2]. Yet, whether the load effect on selective attention is domain-general or domain-specific remains unresolved.

### The domain-general control account

According to the domain-general account, memory load on one content domain can affect the control of attention in another content domain. That is, attentional control supervises selection regardless of information domain. de Fockert and his colleagues [1] provided the first direct evidence for the domain-general effect of memory load on selective attention using a dual-task paradigm. They asked participants to maintain digits while classifying the target name as a pop star or a politician and ignoring a distractor face that was either a pop star or a politician. The results showed that activity related to face processing in the fusiform gyrus and extrastriate visual cortex was significantly greater under high digit load than under low digit load. Memory load on the phonological domain impairs the control of distractor processing in the visuospatial domain.

Lavie and her colleagues [2], [3] proposed a cognitive load theory to explain the role of working memory in selective attention. They suggested that memory load plays a key role in maintaining processing priority, such that more distractor interference would occur under high working memory load than under low load because there would be less cognitive resources to separate target- and distractor-related information. Lavie et al. [2] manipulated memory load by varying the number of digits to be maintained in working memory while the participants performed a letter flanker task. The results showed that distractor interference was greater in the high-digit-load condition than in the low-digit-load condition. Kelley and Lavie [3] asked participants to maintain digits in working memory while performing a categorization task on object images and ignoring a distractor object. They found that the distractor-elicited activation in visual areas (V1, V2, V3/Vp, and V3a/V4v) was higher under high working memory load than under low working memory load. That is, maintaining more information in working memory interrupts the ability to actively control and focus attention on the task-relevant target.

A similar effect of working memory load increasing distractor processing has been shown in various task contexts. For example, a task-irrelevant color singleton delayed shape search when working memory was loaded with digits [4]. The magnitude of the Ebbinghaus illusion, a circle is perceived as larger when it is surrounded by large circles than when it is surrounded by smaller circles, was larger when participants had to maintain a large digit set in working memory than when maintaining a small set [5]. Rissman, Gazzley, and D'Esposito [6] found that the distractor-elicited activation in areas related to face or scene processing was greater under high digit load (presented in the auditory modality) than under low digit load. They suggested that these findings support a domain-general effect because digits are not in the same domain as faces or scenes. In an inattentional blindness paradigm [7], participants were shown a brief display and asked to judge whether a horizontal or a vertical line of a cross was longer while maintaining digits in working memory. An unexpected geometrical feature was presented in a critical trial. The results showed that more participants detected the unexpected stimuli under high load than under low load. These findings suggest that the effect of working memory load on selective attention is more likely to be domain-general than domain-specific [7], [8].

### The specialized load account

Although evidence from a range of studies and measures suggests a domain-general effect of memory load on selective attention, several studies have found that the effect of working memory load on distractibility is domain-specific. That is, memory load influenced control of distractor processing only when the memory task and the attention task demand resources in the same content domain. Selective attention operates in a specific content domain that can be independent of memory load in another domain. When a search task required the participants to indicate the direction of a gap in a rectangular or the location of a target shape in the search array [9]–[11], visual search remained efficient under high color or object load [10], [11] and was delayed when working memory was occupied by visuospatial information [9], [11]. Lavie and de Fockert [4] suggested that these findings may have resulted from not using a salient competing distractor. They used a singleton color distractor in a visual search task and found that the distractibility increased to a greater extent under high digit load than under low or no load. Their results suggest a domain-general effect because load on the phonological domain impairs control of distractor processing in the color domain. However, Burnham and his colleagues [12] used a similar paradigm and found a domain-specific effect. They used a singleton search task involving spatial arrangement of color stimuli with a concurrent working memory (color, spatial, and phonological) task. High load led to a larger distractibility effect than low load in the color and spatial load conditions, but not in the phonological load condition. The effect of memory load on distractibility is domain-specific.

The studies using a non-search task showed that whether high working memory load increases or reduces distractibility depends on whether the contents of the working memory task overlap with the to-be-attended or to-be-ignored feature in a selective attention task, respectively [13], [14]. Under a high letter load, the irrelevant color of a Stroop color word interfered with word naming to a greater extent compared with the no-load condition. When color was the attended feature, the interference caused by the word was reduced. Moreover, a high load of maintaining spatial locations had no effect on distractor processing in either case [14]. Similarly, when the working memory task involved memorizing either faces or houses and when the selective attention task also required attending to faces while ignoring houses (or vice versa), distractor effects increased when the items maintained in working memory were in the same category as the targets of the attention task, and declined when they were in the same domain as the distractors [13]. Maintaining information in working memory impairs processing of same-domain information. They suggested that distractibility increases when the type of working memory load overlaps with target processing. When target processing and the contents of working memory do not overlap, distractibility remains constant regardless of the load in working memory. When the contents in working memory and the ignored information are in the same domain, distractor processing declines.

de Fockert [8] argued that Kim et al.'s view [13], [14] is not supported when the to-be-attended feature and the to-be-ignored feature are in the same domain. According to Kim et al.'s [13], [14] suggestion, the overlap between the contents of working memory and target information increases distractor processing, while the overlap between the contents of working memory and distractor information reduces distractor processing. The two forces should counteract each other and distractibility should be eliminated when the contents of working memory overlap with both the target and the distractor information in the same domain. Yet, previous studies using this experimental context showed greater distractor processing under high load [2], [15]. Thus, it is unclear whether cognitive control of processing priority in selective attention is domain-general or domain-specific when working memory contents overlap domains with both the target and distractor information. The current study is designed to clarify this issue by orthogonally manipulating the types of representations that are activated by a memory task and by a selective attention task.

### Sources of inconsistency

A close examination of the experimental contexts adopted in previous studies showed that two factors might have caused inconsistent findings: materials used in the memory task and the attention task, and the index used to determine the effect of memory load on selective attention. The findings that support a domain-general effect may have arisen because the two tasks share content representations in the same domain. Digits and letters [1], [2] involve phonological representations [16], [17]. Faces of familiar pop stars or politicians [1] activate corresponding name codes, and face and scene categorization [6] may also engage subvocal naming of the category. In contrast, the results supporting a domain-specific effect [12]–[14] may have arisen because of feature overwriting. For example, the memorized letters share features with the Stroop words [14], memorized colors occur in the search display in about half of the trials [12], and memorized faces or scenes share many features with the faces or scenes in the attention task [13]. Oberauer [18] showed that interference between storage and processing in working memory occurred when the stimuli used in the two tasks contained phonologically overlapping features. When stimuli did not share phonological features, processing did not impair memory performance.

The index used to determine the effect of memory load on selective attention differs between the studies that show a domain-general effect and the studies that show a domain-specific effect. The studies that showed a domain-general effect [1]–[4], [6] focused on the effect of memory load on distractor processing so that the measure could provide direct evidence for assessing the effects of differential loads on control of selection. The studies that showed a domain-specific effect [9]–[14] compared performance between a dual task and a single task. The domain-specific effect may have arisen from dual-task costs due to the difficulty of coordinating two tasks [16], [19] that share representations in the same domain.

### The Current Study

The purpose of the current study is to investigate whether cognitive control of processing priority in selective attention demands domain-general or domain-specific resources. We adopted the flanker task to reflect the effectiveness of distractor exclusion because this task requires participants to select a task-relevant target into the focus of attention while excluding a distractor. When the distractor activates a response that is incompatible with target selection, distractor exclusion is in demand to activate frontal regions [20], [21]. Thus, distractor exclusion reflects attentional control that operates on competing representations to prioritize target processing in selective attention. We used a dual-task paradigm and orthogonally manipulated the type of representation that must be maintained in working memory and the type of representation upon which distractor exclusion must be performed. To reduce feature overwriting, we used stimuli that share few features for the two tasks. Rather than comparing the performance between a single-task and a dual-task condition that may reflect dual-task coordination cost, we focused on distractor interference under low and high working memory load.

In Experiment 1a, we used a digit memory task with a letter flanker task as adopted in Lavie et al.'s [2] study. By using different features in the memory task and attention task, we can exclude the possibility of the feature-overwriting effect. In Experiment 1b, the maintained representations involved objects that are difficult to verbally code. In Experiment 2, we presented auditory digits with a letter flanker task to exclude the involvement of resource competition within the same input modality because visual and auditory information is processed in separate neural regions [22]. In Experiments 3a and 3b, we replaced the letter flanker task with an object flanker task while manipulating the memory load of object and digit representation, respectively. According to the cognitive load theory, we expect a domain-general effect. In contrast, we expect a domain-specific effect according to Kim et al.'s specialized load account [13], [14] if the increase in distractibility is greater than the reduction of distractibility when the contents of working memory overlap with both the target and distractor representations in the same domain.

## Experiment 1

In this experiment, we adopted the method that was used in the first experiment conducted by Lavie et al. [2]. We used digits in the memory task and letters in the flanker task in Experiment 1a. In the digit memory task, the participants memorized six digits and one digit in the conditions of high- and low-working memory load, respectively. In the letter flanker task, the participants were asked to identify whether the target letter was either an *X* or an *N*. In Experiment 1b, we used nonverbalized objects in the memory task. In the object memory task, four objects were presented in the condition with high working memory load because previous studies showed that visual working memory has a maximum capacity of four objects [23]–[27] and can have only one object with no categorical information [28]. Furthermore, the participants in a pilot experiment showed chance-level performance when memory set size was more than four objects.

### Method

#### Ethical Statement

This study was approved by the Research Ethics Office of National Taiwan University Committee. Written consent was obtained from all participants in advance of their participation, which included appropriate information to ensure informed consent and the right to withdraw without penalty.

#### Participants

Forty-eight individuals participated in the experiment in exchange for partial course credit. Half of the participants volunteered in Experiment 1a, and the other half of the participants volunteered in Experiment 1b. All of the participants had normal or corrected-to-normal vision, were between 18 and 26 years old (*M* = 20.7, *SD* = 1.9) and were unaware of the purpose of the experiment.

#### Apparatus

The experiment was performed using E-Prime software (Psychology Software Tool Inc.) on a 17-inch CRT monitor with a vertical refresh rate of 60 Hz connected to a PC with a 3.40 GHz Intel Pentium IV processor. The participants viewed the stimuli with their head resting on a chin rest at a distance of 60 cm.

#### Design

The experiment followed a 2 (working memory load: high and low) x 3 (distractor compatibility: compatible, incompatible, and neutral) within-subject factorial design. Each participant completed 288 trials, such that each cell contained 48 observations. The factor of load manipulation consisted of four blocks (two for the high-load condition and two for the low-load condition) with 72 trials in each block. Distractor compatibility was randomized within each block.

#### Stimuli

**Figure 1:**
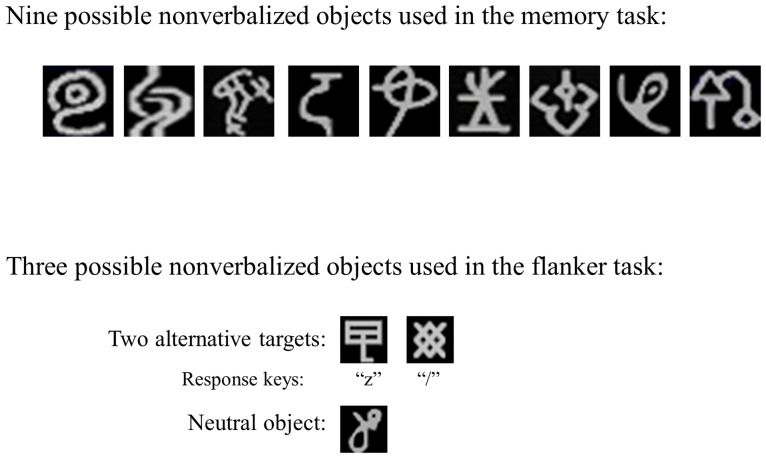
The top panel shows nonverbalized objects used in the memory task in both Experiment 1b and Experiment 3a. The bottom panel shows nonverbalized objects used in the flaker task in Experiment 3.

All stimuli were light gray. In the condition with high working memory load for the digit memory task, the digit display consisted of six digits, each subtending a visual angle of 0.58° horizontally and 1.08° vertically. These digits were presented equally spaced in a horizontal row that subtended 4.45° from edge to edge. In the low-load condition, only one digit was presented on the screen center. For the masking array, one or six asterisks were positioned at the locations of the digits in the low- or high-load condition, respectively. The digits in the memory set were chosen at random from 1 to 9, and each digit was equally likely to be present in the memory set of each load condition. The order of six digits in the memory set of the high working memory load was random, with the constraint that no more than two consecutive digits were presented in sequence. For the memory probe, one digit was presented in the center. Probe digits were equally likely to be present or absent in the trial's memory set and were equally likely to probe any of the six digits in the trials of high memory load. In the object memory task in Experiment 1b, the digits were replaced by nonverbalized objects that were chosen from nine possible figures (see Figure 1), with four objects under high load and a single object under low load.

In the letter flanker task, a circular array consisting of one target letter that was either an *X* or an *N* and five nontarget small circles were presented in the center of the display, extending a visual angle of 4.33° in diameter. The circular array was used to ensure that equal distance from each stimulus to the fixation, as used in many studies that studied control of priority in selective attention [29]–[32]. The target letter occurred at each of the six locations with equal frequency. A letter outside the circular array is defined as the distractor. The peripheral distractor was placed 3.29° from the array on the horizontal meridian in the right or left field with equal frequency. The target letter subtended 0.43° horizontally and 0.54° vertically. A distractor letter subtended 0.54° horizontally and 0.66° vertically and was equally assigned to be compatible (e.g., an *X* when the target was an *X*), incompatible (e.g., an *X* when the target was an *N*), or neutral (the letter *L*).

#### Procedure

The presentation order alternated between high working memory load and low working memory load. Half of the subjects began with a high working memory load block (high load → low load → high load → low load), and the other half began with a low working memory load block (low load → high load → low load → high load). Four experimental blocks were used, preceded by two blocks of 10 practice trials from each load condition, which were presented in the same order as in the experimental blocks.

**Figure 2:**
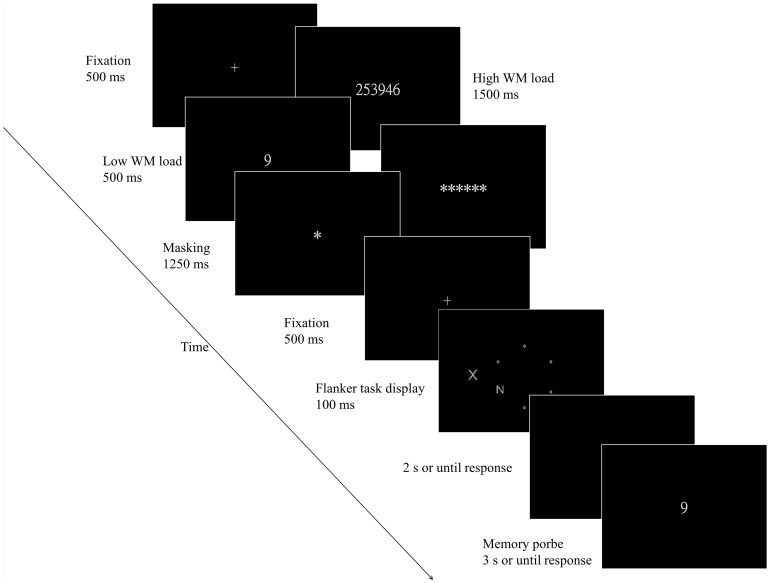
Examples of the trial procedure used in Experiment 1a.

Each trial began with a fixation cross in the center of the screen for 500 ms, followed by a memory set that was presented for 500 ms in the low-load condition or for 1500 ms in the high-load condition. A masking array was then presented for 1250 ms in both the low and high working memory load conditions. The masking array was followed by a fixation point presented for 500 ms and was replaced with brief presentation (100 ms) of the letter flanker task display. The participants were required to respond using their right hand to press *“Z”* on the keyboard if the target letter on this display was an *X* or using their left hand to press *“/”* if the target was an *N*. The participants were encouraged to ignore the distractor to avoid the potentially disruptive effect. When the participants responded (or after a 2 s elapse with no response), a memory probe was presented and remained on the display until a response was given (or for 3 s with no response) by pressing *“X”* on the keyboard to indicate that the probe digit (object) was present in the trial's memory set or by pressing *“.”* to indicate that the probe digit (object) was absent from the trial's memory set. A sticker with labelling was glued on each response button so that the participants would not be interfered by the original functions of the buttons. A 500-ms auditory tone immediately followed incorrect responses and was also presented if subjects failed to respond to either task within the given time window. Figure 2 shows examples of the procedure for each condition.

### Results

Tables 1 and 2 show the percentage correct and median reaction time (RT) for recognizing memory probes in each condition. To reflect memory accurately stored in working memory, correction for guessing is required [33]. We subtracted the false alarm rate (respond “present” when the digit was absent in the memory set) from the hit rate (respond “present” when the digit was in the memory set) of each condition to analyze accuracy data. Memory performance was analyzed using a 2 (working memory load: high and low) x 3 (distractor compatibility: compatible, incompatible, and neutral) repeated-measures analysis of variance (ANOVA) on corrected recognition accuracy and median RTs of correct responses (including hit trials and correct rejection trials). For the flanker task performance, trials were included for accuracy analysis only when memory judgment was correct, because memory errors could result from not maintaining information in working memory and the effect of maintenance on selective attention is the issue of concern. RT analysis only included trials when memory judgment was correct, flanker performance was correct, and RT was longer than 100 ms but shorter than 2 s. Given that our interest is on distractibility that has been indexed by the contrast in neural activation [20],[21] or behavioral performance [34] between compatible and incompatible trials, we also used a 2 (working memory load: high and low) x 2 (distractor compatibility: compatible and incompatible) repeated-measures ANOVA to analyze flanker performance. We present the results of Experiments 1a and 1b separately.

**Table 1:** Mean Percentage of Accuracy on Judging Memory Probes after Neutral (N), Compatible (C), and Incompatible (IC) trials.

		High Working Memory Load						Low Working Memory Load					
		Present			Absent			Present			Absent		
		C	IC	N	C	IC	N	C	IC	N	C	IC	N
Experiment 1a	Mean	95.6	95.8	95.4	97.7	95.2	95.8	94.0	95.8	94.8	95.8	95.8	95.5
	SE	1.0	1.2	1.1	0.6	1.3	1.0	1.5	0.9	0.9	0.7	1.4	1.0
Experiment 1b	Mean	73.0	70.9	70.9	83.8	83.8	82.2	93.7	92.4	92.9	95.6	95.3	96.2
	SE	3.4	3.0	2.7	2.7	2.2	2.4	1.4	1.6	1.3	1.0	0.8	0.9
Experiment 2	Mean	93.4	93.3	94.8	94.6	96.6	98.0	95.2	96.3	97.4	96.6	97.0	96.0
	SE	1.7	1.3	1.4	1.1	0.8	0.7	0.9	1.1	0.9	0.8	0.6	1.1
Experiment 3a	Mean	63.8	64.8	64.2	79.3	78.6	75.1	91.9	89.8	89.9	91.6	90.8	91.3
	SE	3.2	3.2	3.4	2.9	2.6	3.4	2.3	2.0	1.8	2.2	1.8	1.9
Experiment 3b	Mean	95.6	94.0	95.4	95.6	96.4	96.6	93.2	92.5	93.6	95.5	95.4	96.3
	SE	1.5	1.1	1.2	1.0	0.8	1.0	1.6	1.4	1.2	1.3	1.2	1.0

**Table 2:** Average of Median RTs (ms) to the Memory Probes after Neutral (N), Compatible (C), and Incompatible (IC) trials.

		High Working Memory Load			Low Working Memory Load		
		C	IC	N	C	IC	N
Experiment 1a	Mean	851.6	867.8	862.1	744.5	759.6	749.1
	SE	43.6	43.5	42.3	44.2	43.4	43.9
Experiment 1b	Mean	979.5	993.9	987.5	730.4	755.7	747.4
	SE	36.8	35.2	35.2	28.5	29.5	28.5
Experiment 2	Mean	899.7	908.8	908.3	654.9	680.5	669.7
	SE	37.7	38.1	36.6	31.2	30.9	28.6
Experiment 3a	Mean	893.1	897.6	907.1	708.3	714.8	714.4
	SE	34.6	33.8	35.6	29.6	26.1	28.9
Experiment 3b	Mean	877.6	880.3	856.3	720.7	731.6	725.2
	SE	38.2	35.6	34.4	31.8	31.9	31.7

#### Experiment 1a

*Digit memory task.* The results showed a significant effect of working memory load on RTs [*F*(1, 23)  = 19.62, *p*<.001, η_p_^2^ = .46] with a faster RT in the low-load condition (*M* = 709.80 ms, *SE* = 15.3) than in the high-load condition (*M* = 827.23 ms, *SE* = 20.2). This finding confirmed that our manipulation of memory set size was effective for increasing memory load. No other effects were significant (*ps*>.17). The analysis of corrected recognition showed null effects (*ps*>.59).

*Letter flanker task.* The number of observations in each cell ranged from 36 to 48 trials across the participants. The results (see Table 3) showed a significant effect of distractor compatibility [*F*(1, 23)  = 17.66, *p*<.001, η_p_^2^ = .43], with a faster RT in the compatible condition (*M*814.0 ms, *SE* = 22.0) than in the incompatible condition (*M* = 837.9 ms, *SE* = 23.2). There was no significant effect of memory load (*p* = .52), but its interaction with distractor compatibility was significant [*F*(1, 23)  = 6.29, *p* = .019, η_p_^2^ = .21]. Although a simple main effect analysis showed that the distractor compatibility effect was significant in both the low-load [*F*(1, 46)  = 9.30, *p* = .004, η_p_^2^ = .17] and high-load conditions [*F*(1, 46)  = 23.20, *p*<.001, η_p_^2^ = .34], the effect size in the high-load condition was larger than in the low-load condition. Accuracy data (see Table 4) showed a significant distractor compatibility effect [*F*(1, 23)  = 4.49, *p* = .043, η_p_^2^ = .19] with better performance in the compatible condition than in the incompatible condition. No other effects reached significance in the accuracy data (*ps*>.14).

**Table 3:** Mean RTs (ms) to the Flanker Task for Neutral (N), Compatible (C), and Incompatible (IC) trials under Each Load Condition.

		High Working Memory Load			Low Working Memory Load		
		N	C	IC	N	C	IC
Experiment 1a	Mean	854.6	807	892.4	848.2	821.1	875.3
	SE	34.1	31.1	38.5	34.9	31.8	36.4
Experiment 1b	Mean	813.2	794.1	846.4	804.1	778.3	833.1
	SE	33.1	28	33.3	26.8	28.1	32
Experiment 2	Mean	755.6	707.9	793.9	750.6	711.4	761.7
	SE	46.6	45.7	47.4	45.5	44.1	44.7
Experiment 3a	Mean	802.3	764.8	813.8	774.1	762.7	785.6
	SE	24.7	24.6	25.1	27.3	28.6	28.6
Experiment 3b	Mean	782.5	782.8	804.0	828.7	802.5	830.8
	SE	19	24.1	21.8	29.7	28	28.8

**Table 4:** Mean Percentage of Correct Responses on the Flanker Task for Neutral (N), Compatible (C), and Incompatible (IC) Trials under Each Load Condition.

		High Working Memory Load			Low Working Memory Load		
		N	C	IC	N	C	IC
Experiment 1a	Mean	98.5	97.5	96.8	97.7	97.1	96.9
	SE	0.4	0.8	0.7	0.6	0.7	0.6
Experiment 1b	Mean	98.4	98.4	95.8	98.4	97.5	96
	SE	0.4	0.4	0.8	0.4	0.7	0.8
Experiment 2	Mean	98.9	97.6	96.4	98.5	98	95.6
	SE	0.5	0.8	1	0.6	0.5	0.8
Experiment 3a	Mean	96.3	98.0	96.1	97.1	96.4	96.0
	SE	0.8	0.6	0.8	0.6	0.7	0.7
Experiment 3b	Mean	89.8	88.6	88.2	94.6	95.7	93.6
	SE	2.4	2.2	2.6	1.4	1	1.5

#### Experiment 1b

*Object memory task.* The results (see Table 2) showed a significant effect of working memory load on RTs [*F*(1, 23)  = 153.80, *p*<.001, η_p_^2^ = .87], with a faster RT in the low-load condition (*M* = 744.50 ms, *SE* = 16.5) than in the high-load condition (*M* = 986.97 ms, *SE* = 20.4). The main effect of distractor compatibility and its interaction with working memory load were not significant (*ps*>.06). Analysis of corrected recognition showed a significant effect of memory load [*F*(1, 23)  = 63.37, *p*<.001, η_p_^2^ = .73], with better performance in the low-load condition (*M* = 88.69%, *SE* = .94) than in the high-load condition (*M* = 54.79%, *SE* = 2.30). These findings confirm that our manipulation of memory set size was effective for increasing memory load. The main effect of distractor compatibility and its interaction with working memory load were not significant (*ps*>.48).

*Letter flanker task.* The number of observations in each cell ranged from 27 to 48 trials across the participants. The results (see Table 3) showed a significant main effect of distractor compatibility [*F*(1, 23)  = 12.63, *p* = .002, η_p_^2^ = .35], with a faster RT in the compatible condition (*M* = 786.21 ms, *SE* = 19.7) than in the incompatible condition (*M* = 839.74 ms, *SE* = 22.9).The main effect of working memory load and its interaction was not significant (*ps*>.41). Accuracy data (see Table 4) showed a significant effect only for distractor compatibility [*F*(1, 23)  = 9.64, *p* = .005, η_p_^2^ = .29], whereas others showed a null effect (*ps*>.26). To assess the strength of evidence for the null interaction effect in the RT data, we estimated the posterior probability and Bayes factor to verify the likelihood of the null hypothesis given the data under the assumption of equal prior probabilities between the null and the alternative hypotheses [35]. The Bayes factors and posterior probabilities of the null hypothesis for the interaction were 4.757 and.826, respectively. A posterior probability in the range of.75 to.95 shows positive evidence [36] in support for the null interaction hypothesis over the alternative hypothesis of a significant effect.

**Figure 3:**
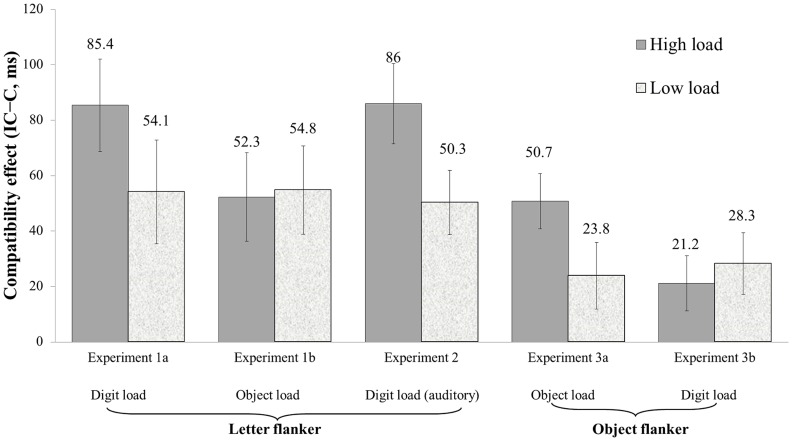
Compatibility effects (incompatible RT − compatible RT) under each condition for the three experiments.

To test whether the interference relies on the same domain, we used RT data to conduct a 2 (experiment: same domain and different domain) x 2 (working memory load: high and low) x 2 (distractor compatibility: compatible and incompatible) mixed-design comparison. A significant three-way interaction was observed [*F*(1, 46)  = 4.24, *p* = .043, η_p_^2^ = .08]. A simple main effect analysis showed that the compatibility effect that was indexed by better performance in the compatible condition than in the incompatible condition was significant in all conditions: the high-load condition in Experiment 1a [*F*(1, 92)  = 25.61, *p*<.001, η_p_^2^ = .22], the low-load condition in Experiment 1a [*F*(1, 92)  = 10.27, *p* = .002, η_p_^2^ = .10], the high-load condition in Experiment 1b [*F*(1, 92)  = 9.58, *p* = .003, η_p_^2^ = .09], and the low-load condition in Experiment 1b [*F*(1, 92)  = 10.53, *p* = .002, η_p_^2^ = .10]. However, the effect size in the high-load condition in Experiment 1a was greater than in the other conditions. Figure 3 shows the degree of distractor interference in each condition.

### Discussion

The results showed a domain-specific effect of memory load on distractibility. The effectiveness of distractor exclusion was reduced when a high demand was placed on storing representations in the same verbal domain. As a result, distractor interference (see Figure 3) was greater under high memory load (*M* = 85.45, *SE* = 16.7) than under low memory load (*M* = 54.11, *SE* = 18.7) only in Experiment 1a, in which digits and letters are both represented in the verbal domain. In contrast, the magnitude of distractor interference under high load in Experiment 1b (*M* = 52.27, *SE* = 16.0) was not significantly different from the magnitude of distractor interference under low load (*M* = 54.80, *SE* = 15.9) when storage required maintaining nonverbalized objects in working memory. More importantly, the magnitude of distractor interference was similar between the two experiments under low memory load. Distractibility was unaffected by the manipulation of representation domain when the demand on working memory was low. Increased distractibility emerges because of loading too many items in the same content domain.

## Experiment 2

The results of Experiment 1 showed a domain-specific effect of memory load on distractibility. The larger compatibility effect under high load in Experiment 1a may have resulted from allocating more resources to maintaining digits in working memory for accurate performance under high load. This result may have occurred because the digits were presented visually such that they activated the representation of both visual (image) and verbal (phonemic and semantic) codes. Thus, we presented auditory digits in this experiment to eliminate the role of input modality, given that modality-specific capacity in neural activity has been observed for auditory and visual information [22].

Another reason for conducting this experiment was the inconsistency observed in two studies that investigated the effect of process operation on memory representations using auditory presentation. Rissman et al. [6] found that high auditory memory load leads to more processing of irrelevant visual images, showing a modality-general effect. In contrast, Jarrold et al. [17] found that spatial symmetry judgments of visual letters compared with verbal rhyming judgments of visual letters caused less deficit on an auditory letter memory task. This finding suggests a domain-specific effect in the interaction between storage and control of processing priority in selective attention. We conducted this experiment to examine whether the effect of a high verbal working memory load on distractor exclusion in the letter flanker task is domain-general or domain-specific.

### Method

#### Participants

A total of 20 individuals participated in the experiment in exchange for partial course credit. All participants had normal or corrected-to-normal vision, were between 19 and 24 years old (*M* = 21.3, *SD* = 1.4), and were unaware of the purpose of the experiment.

#### Apparatus, design, stimuli and procedure

All aspects were similar to Experiment 1a except that the digits were presented in auditory modality. The audio files were recorded from a male speaker, with a separate file for each digit (1 to 9). In the high-load condition, six digits were presented sequentially with the duration of 250 ms for each, yielding a total duration of approximately 1500 ms.

### Results

The cutoff points were the same as those used in Experiment 1. The data for the memory task were analyzed using a 2 (working memory load: high and low) x 3 (distractor compatibility: compatible, incompatible, and neutral) repeated-measures ANOVA. Flanker task performance was analyzed using a 2 (working memory load: high and low) x 2 (distractor compatibility: compatible and incompatible) repeated-measures ANOVA.

*Digit memory task.* The results (see Tables 2) showed a significant effect of working memory load on RTs [*F*(1, 19)  = 107.85, *p*<.001, η_p_^2^ = .85], with a faster RT in the low-load condition (*M* = 668.36 ms, *SE* = 17.2) than in the high-load condition (*M* = 905.60 ms, *SE* = 21.3). The main effect of distractor compatibility and its interaction with memory load were not significant (*ps*>.14). Analysis of corrected recognition showed a significant effect of memory load [*F*(1, 19)  = 6.30, *p* = .020, η_p_^2^ = .25], with better performance in the low-load condition (*M* = 94.45%, *SE* = .76) than in the high-load condition (*M* = 91.02%, *SE* = 1.06). The main effect of distractor compatibility and its interaction with memory load were not significant (*ps*>.07). These findings confirm that our manipulation of memory set size was effective for increasing memory load.

*Letter flanker task.* The number of observations in each cell ranged from 36 to 48 trials across the participants. The results (see Tables 3 and 4) showed a significant effect of distractor compatibility [*F*(1, 19)  = 46.87, *p*<.001, η_p_^2^ = .71], with a faster RT in the compatible condition (*M* = 709.67 ms, *SE* = 31.4) than in the incompatible condition (*M* = 777.80 ms, *SE* = 32.3). There was no significant effect of memory load (*p* = .25), although its interaction with distractor compatibility was significant [*F*(1, 19)  = 4.35, *p* = .048, η_p_^2^ = .19]. A simple main effect analysis showed that the compatibility effect was significant in both the low-load [*F*(1, 38)  = 17.71, *p*<.001, η_p_^2^ = .20] and high-load conditions [*F*(1, 38)  = 42.95, *p*<.001, η_p_^2^ = .74]; the effect size in the high-load condition (*M* = 85.96, *SE* = 14.5) was larger than that in the low-load condition (*M* = 50.30, *SE* = 11.6). Accuracy data showed a significant compatibility effect [*F*(1, 19)  = 4.92, *p* = .037, η_p_^2^ = .20], and no other effects reached significance (*ps*>.4).

### Discussion

The results are consistent with those observed in Experiment 1a. High verbal working memory load increased distractibility in the letter flanker task. This finding excludes the possibility that the lower effectiveness of distractor exclusion under high digit load observed in Experiment 1a resulted from resource consumption in both the visual and verbal domains. The influence of a high digit load in working memory on distractor processing is domain-specific even when the storage task and the attention task demand processing in separate input modalities. It is representation proximity that impairs the effectiveness of distractor exclusion under high load. Representation proximity arises from sharing phonological codes [16], [17] and from being integrated into symbols that are encountered frequently in everyday activities (e.g., the designated location of a parking space). Subvocal categorization of faces or scenes may be a plausible reason for observing a domain-general effect in Rissman et al.'s [6] study.

## Experiment 3

The results of Experiments 1 and 2 showed that high working memory load leads to greater distractor interference only when the two tasks competed for resources in the same domain (Experiments 1a and 2) but not when they demanded resources in different domains (Experiment 1b). However, the results may be specific to the use of the letter flanker task. Moreover, memory performance under high working memory load is low in Experiment 1b compared with performance in Experiments 1a and 2. The null results of Experiment 1b could be a consequence of not remembering objects in working memory, thereby not affecting the concurrent attention task. The contrast may not have anything to do with the difference in domain per se.

To examine whether the results are specific to the use of a letter flanker task or arise from the discrepancy in difficulty between different domains of load manipulation, we used an object flanker task with an object memory task (Experiment 3a) and a digit memory task (Experiment 3b). If the results show a domain-specific effect of working memory load on distractor exclusion, then the finding should corroborate the domain-specific interaction between memory maintenance and control of processing priority. If the findings of Experiments 1 and 2 had resulted from the discrepancy in difficulty between the two load manipulations, participants would not remember the objects and object load should not modulate the compatibility effect. Instead, the remembering of digits should modulate the degree of distractor interference.

### Method

#### Participants

A total of 48 individuals participated in the experiment in exchange for partial course credit. Half of the participants volunteered in Experiment 3a, and the other half volunteered in Experiment 3b. All participants had normal or corrected-to-normal vision, were between 19 and 29 years old (*M* = 20.8, *SD* = 1.9), and were unaware of the purpose of the experiment.

#### Apparatus, design, stimuli and procedure

All aspects were similar to Experiment 1, except for three changes. First, we used nonverbalized objects in the flanker task (see Figure 1). Second, to ensure that the participants were familiar with the mapping between object stimuli and response keys before the formal test, there was a response mapping task containing 30 trials in which a target object was presented and the participants were asked to press a response key as quickly and accurately as possible. Third, to assure that the participants could identify the objects more precisely, the target object was enlarged to a visual angle of 0.62° horizontally and vertically, and the distractor object was enlarged to a visual angle of 0.77° horizontally and vertically.

### Results

The cutoff points were the same as those used in Experiment 1. The data for the memory task were analyzed using a 2 (working memory load: high and low) x 3 (distractor compatibility: compatible, incompatible, and neutral) repeated-measures ANOVA. Flanker task performance was analyzed using a 2 (working memory load: high and low) x 2 (distractor compatibility: compatible and incompatible) repeated-measures ANOVA. We present the results of Experiments 3a and 3b separately.

#### Experiment 3a

*Object memory task.* The results (see Table 2) showed a significant effect of memory load on RTs [*F*(1, 23)  = 92.89, *p*<.001, η_p_^2^ = .80], with a faster RT in the low-load condition (*M* = 715.28 ms, *SE* = 16.3) than in the high-load condition (*M* = 911.67 ms, *SE* = 18.7). The other effects were not significant (*p*s>.12). Analysis of corrected recognition showed a significant effect of memory load [*F*(1, 23)  = 138.74, *p*<.001, η_p_^2^ = .86], with better performance in the low-load condition (*M* = 83.41%, *SE* = 1.7) than in the high-load condition (*M* = 43.3%, *SE* = 2.6). The main effect of distractor compatibility and its interaction with memory load were not significant (*ps*>.11). These findings confirm that our manipulation of memory set size was effective for increasing memory load.

*Object flanker task.* The number of observations in each cell ranged from 26 to 48 trials across the participants. The results (see Table 3) showed a significant effect of distractor compatibility [*F*(1, 23)  = 16.26, *p* = .001, η_p_^2^ = .41], with a faster RT in the compatible condition (*M* = 754.86 ms, *SE* = 17.5) than in the incompatible condition (*M* = 792.15 ms, *SE* = 17.9). There was no main effect of working memory load (*p* = .482), but its interaction with distractor compatibility was significant [*F*(1, 23)  = 5.00, *p* = .033, η_p_^2^ = .18]. Although a simple main effect analysis showed that the compatibility effect was significant in both the low-load [*F*(1, 46)  = 4.67, *p* = .034, η_p_^2^ = .09] and high-load conditions [*F*(1, 46)  = 21.15, *p*<.001, η_p_^2^ = .31], and the effect size in the high-load condition was larger than in the low-load condition. Accuracy data (see Table 4) showed null effects (*ps*>.06).

#### Experiment 3b

*Digit memory task.* The results (see Table 2) showed a significant effect of working memory load on RTs [*F*(1, 23)  = 16.43, *p*<.001, η_p_^2^ = .42], with a faster RT in the low-load condition (*M* = 725.86 ms, *SE* = 18.10) than in the high-load condition (*M* = 871.40 ms, *SE* = 20.6). This finding confirms that our manipulation of memory set size was effective for increasing memory load. The main effect of distractor compatibility and its interaction with working memory load were not significant (*ps*>.24). Analysis of corrected recognition showed null effects (*ps*>.22).

*Object flanker task.* The number of observations in each cell ranged from 30 to 48 trials across the participants. The results (see Table 3) showed a significant effect of distractor compatibility [*F*(1, 23)  = 7.38, *p* = .012, η_p_^2^ = .24], with a faster RT in the compatible condition (*M* = 792.68 ms, *SE* = 18.3) than in the incompatible condition (*M* = 817.42 ms, *SE* = 18.0). The main effect of memory load and its interaction with distractor compatibility were not significant (*ps*>.45). The Bayes factors and posterior probabilities of the null statistical hypothesis for the interaction were 3.925 and.797, respectively. This finding provides positive evidence in support for the null hypothesis over the alternative hypothesis. Accuracy data (see Table 4) showed a significant effect of memory load [*F*(1, 23)  = 7.07, *p* = .013, η_p_^2^ = .24], with a higher accuracy in the low-load condition (*M* = 94.7%, *SE* = 0.90) than in the high-load condition (*M* = 88.4%, *SE* = 1.71), whereas the others showed null effects (*ps*>.28).

To test whether distractor interference under high working memory load was the consequence of sharing resources in the same content domain, we used RT data to conduct a 2 (experiment: same domain and different domain) x 2 (working memory load: high and low) x 2 (distractor compatibility: compatible and incompatible) mixed-design analysis. A significant three-way interaction was observed [*F*(1, 46)  = 4.42, *p* = .039, η_p_^2^ = .09]. A simple main effect analysis showed a significant compatibility effect in all conditions: high load in Experiment 3a [*F*(1, 92)  = 22.04, *p*<.001, η_p_^2^ = .19], low load in Experiment 3a [*F*(1, 92)  = 4.868, *p* = .028, η_p_^2^ = .05], high load in Experiment 3b [*F*(1, 92)  = 3.86, *p* = .050, η_p_^2^ = .04] , and low load in Experiment 3b [*F*(1, 92)  = 6.84, *p*<.010, η_p_^2^ = .07]. However, the effect size in the high-load condition of Experiment 3a was greater than that in the other three conditions. The results revealed that whether two tasks compete for resources in the same or different content domains modulates the degree of distractor interference under high working memory load.

### Discussion

The results of Experiment 3 further confirm the domain-specific effect of working memory load on distractibility. Distractor interference under high working memory load was greater than under low working memory load only when the two tasks competed for resources in the same content domain. In Experiment 3a, in which we used object memory and object flanker tasks (competing for the same domain of resources), the results showed a larger compatibility effect under high load (*M* = 48.94, *SE* = 10.0) than under low load (*M* = 22.91, *SE* = 11.9). When we used a digit memory task and an object flanker task (resource competition in different domains) in Experiment 3b, the compatibility effect under high load (*M* = 21.22, *SE* = 10.0) was not significantly different from the effect under low load (*M* = 28.26, *SE* = 11.1). The results were inconsistent with the prediction of a domain-general account. The results also refute the suggestion that the domain-specific effects observed in Experiments 1 and 2 arise from differential degrees of difficulty in load manipulation because the null modulation effect of working memory load manifests in Experiment 3b with better memory performance compared with Experiment 3a.

The mean compatibility effect (*M* = 30.33) in the object flanker task was much smaller than the effect (*M* = 61.79) in the letter flanker task observed in Experiments 1 and 2. This difference may have arisen because the nonverbalized objects are unfamiliar; thus, the stimulus-response mapping rule would be less stable. Despite the difference in the magnitude of the compatibility effect, the pattern of results is consistent with that obtained from a letter flanker task. We found no evidence of a domain-general effect in the interaction between memory load and distractor exclusion. The overall RTs under high load were not slower than under low load in the object flanker task. The main effect of load was not significant in either Experiment 3a or Experiment 3b. However, the compatibility effect was the largest under high load when the stimuli of the two tasks belonged to the same domain of object representation. The effect of increasing memory load on distractibility was found to be domain-specific.

## General Discussion

The purpose of this study is to resolve controversy in previous studies by verifying whether control of processing priority in selective attention demands domain-general or domain-specific resources. We interleaved a memory task and a flanker task across three experiments while manipulating the type of representation that must be maintained and the type of representation upon which distractor exclusion must be performed in selective attention. In Experiments 1a and 1b, we interleaved a letter flanker task with memory task that involved digits and nonverbalized objects, respectively. In Experiment 2, we interleaved the memory of auditory digits with a letter flanker task to exclude the involvement of resource competition within the same input modality. In Experiments 3a and 3b, we replaced the letter flanker task with an object flanker task while manipulating memory load on object and digit representation, respectively. The results showed that control of processing priority in selective attention is domain-specific.

The current study is the first attempt to use a dual-task paradigm and orthogonally manipulate the types of representations that are activated by a memory task and by a selection task. Given that digits and letters are represented in proximity within the phonological domain [16], [17], they were used for the phonological stimuli. To eliminate phonological coding, we intentionally used distinctive objects that are difficult to verbalize for the object stimuli. The results consistently showed a domain-specific interaction between memory load and selective attention. The results have important implications for models of attentional control that focus on the effects of cognitive demand on selective attention and for models of working memory that focus on the architecture of cognitive functions.

### Implications for theoretical models

Attentional control is crucial for an individual to select task-relevant information while ignoring task-irrelevant stimuli. Understanding the constraint of attentional control can provide important knowledge for practical issues such as learning, memory, and decision making. We focus on the effect of memory load on attentional control of distractibility in the current study. According to the cognitive load theory [2], the capacity of attentional control for processing priority is reduced under high working memory load, such that high load enhances distractor processing. The cognitive load theory has been integrated into a dual-control model of spatial attention [37]–[39] that suggests both the perceptual load and cognitive load affect spatial focus of attention. When the cognitive load is low, perceptual load dominates the adjustment of spatial focus, with a diffuse and narrow focus under low and high perceptual load, respectively. When the cognitive load is high, cognitive load dominates the adjustment of spatial attention, with a diffuse focus under both low and high perceptual load conditions.

Our results are inconsistent with the prediction of the dual-control model. In our experiments, perceptual load was low while memory load varied between low and high. According to this model, attentional focus should be diffuse in both conditions, such that compatibility effects should be similar between low and high memory load conditions. Our results showed elevated distractibility under high load when the two tasks shared resources in the same domain. One explanation is the differential effects of memory load and cognitive load on attentional control [29]. In the studies that support the dual-control model, the participants must remember the order of the digits. In contrast, our participants did not need to memorize the order. Whether this methodological difference causes the inconsistency remains to be explored for two reasons. First, the participants in Lavie et al.'s [2] study did not need to remember the order and their results are considered as supporting a domain-general effect (for a review, see [7], [8]). Second, the materials used for the memory load task (colored squares) and the cognitive load task (digits) in Konstantinou and Lavie's [17] study may share resources with the secondary shape detection task and with the primary letter search task, respectively. Resource competition between memory of colored squares and shape detection may have led to low sensitivity of the secondary task. In contrast, resource competition between memory of digits and letter search may have deteriorated attentional control and thus the sensitivity of shape detection was better under high load than under low load.

With minor modification, the specialized load account [13], [14] can explain the results of the current study. According to this view, the overlap between the contents of working memory and target information increases distractor processing, while the overlap between the contents and distractor information reduces distractor processing. de Fockert [8] argued that this view is not supported when the to-be-attended feature and the to-be-ignored feature are in the same domain because the two forces should counteract each other when the contents of working memory overlap with both the target and the distractor information in the same domain. Our results showed that distractibility increased in this experimental context, suggesting that the extent of increasing distractibility is greater than the extent of reducing distractibility. That is, the competition for domain-specific resources between memory maintenance and target processing increases the opportunity for the distractor to influence target selection.

The results of the current study also have important implications for models of working memory. In the literature of working memory research, the architecture of cognitive function is a theoretical issue under debate to account for memory forgetting induced by cognitive processing. According to the multiple-component model of working memory [16], [19], [40], working memory consists of two domain-specific slave systems that support storage of visuospatial information and phonological information, respectively. Evidence from the literature of working memory supports this domain-specific view of memory storage using variants of the dual-task paradigm [41]–[43]. In contrast, the time-based resource sharing (TBRS) model suggests that cognitive processing and memory storage compete for limited domain-general attentional resources [44]–[46]. Increasing attentional demand required by a processing task can reduce the resources for refreshing memory representations and hence impair memory storage. Vergauwe et al. [44] provided empirical evidence that showed both verbal and visual-spatial processing influenced memory of verbal and visual-spatial stimuli, reflecting a domain-general effect.

Considering that different materials are often used for different processing tasks, Jarrold et al. [17] used letters for both a verbal processing task that requires rhyming judgments and a visuospatial processing task that demands symmetry judgments along with memory of monosyllable words in dual-task conditions. The results showed both processing tasks impaired memory compared with a baseline single-task condition and the impairment was larger with a verbal processing task than with a visuospatial processing task. That is, both a domain-general effect and a domain-specific effect were observed. They suggested that verbal coding of letters in the symmetry judgment might be a reason for observing the domain-general effect.

The results of the current study did not show a domain-general effect. This finding refutes the TBRS model. If memory storage and cognitive processing compete for domain-general resources as the model postulates, we should have observed comparable degrees of the compatibility effect when memory load increased from low to high across all conditions. Our results are also inconsistent with the observation of a domain-general effect in Jarrold et al.'s [17] study. The inconsistency may have arisen from verbal coding of letters in their study and from the measures used. They used the contrast between a dual-task condition and a single-task condition. In contrast, we compared the degree of distractibility across two dual-task conditions under low- and high-load. According to Baddeley's multiple-component model, coordination of operations between the two domain-specific slave systems is a key function of the central executive. Whereas the domain-general effect in Jarrold et al.'s [17] study may reflect dual-task coordination, the influence of dual-task coordination cost is excluded in our measure.

## Conclusions

Across three experiments in the current study, we manipulated the type of representation that must be maintained in working memory and the type of representation in which distractor exclusion must be performed while searching for a target. The results showed that storage maintenance affects control of processing priority in selective attention in a domain-specific manner. This finding has important implications for the role of working memory in selective attention [2], [13], [14], [29], for models of working memory [16], [19], [45], [46], and may provide directions for future research. Given that working memory and selective attention are two important psychological constructs in cognitive psychology, understanding how these two mechanisms interact in different contexts may reveal the dynamics of cognitive operations in daily activities.

## References

[1] de Fockert JW, Rees G, Frith CD, Lavie N (2001) The role of working memory in visual selective attention. Science 291: 1803–1806. 11230699 10.1126/science.1056496

[2] Lavie N, Hirst A, de Fockert JW, Viding E (2004) Load theory of selective attention and cognitive control. J Exp Psychol Gen 133: 339–354. 15355143 10.1037/0096-3445.133.3.339

[3] Kelley TA, Lavie N (2011) Working memory load modulates distractor competition in primary visual cortex. Cereb Cortex 21: 659–665. 20699229 10.1093/cercor/bhq139 PMC3041013

[4] Lavie N, de Fockert JW (2005) The role of working memory in attentional capture. Psychon Bull Rev 12: 669–674. 16447380 10.3758/bf03196756

[5] de Fockert JW, Wu S (2009) High working memory load leads to more Ebbinghaus illusion. Eur J Cogn Psychol 21: 961–970.

[6] Rissman J, Gazzaley A, D'Esposito M (2009) The effect of non-visual working memory load on top-down modulation of visual processing. Neuropsychologia 47: 1637–1646. 19397858 10.1016/j.neuropsychologia.2009.01.036 PMC2701233

[7] de Fockert JW, Bremner AJ (2011) Release of inattentional blindness by high working memory load: Elucidating the relationship between working memory and selective attention. Cognition 121: 400–408. 21937032 10.1016/j.cognition.2011.08.016

[8] de Fockert JW (2013) Beyond perceptual load and dilution: a review of the role of working memory in selective attention. Front Psychol 4. 10.3389/fpsyg.2013.00287 PMC3659333 23734139

[9] Woodman GF, Luck SJ (2004) Visual search is slowed when visuospatial working memory is occupied. Psychon Bull Rev 11: 269–274. 15260192 10.3758/bf03196569

[10] Woodman GF, Vogel EK, Luck SJ (2001) Visual search remains efficient when visual working memory is full. Psychol Sci 12: 219–224. 11437304 10.1111/1467-9280.00339

[11] Oh S-H, Kim M-S (2004) The role of spatial working memory in visual search efficiency. Psychon Bull Rev 11: 275–281. 15260193 10.3758/bf03196570

[12] Burnham BR, Sabia M, Langan C (2013) Components of working memory and visual selective attention. J Exp Psychol Human: Advance online publication. 10.1037/a0033753 23875574

[13] Park S, Kim M-S, Chun MM (2007) Concurrent working memory load can facilitate selective attention: Evidence for specialized load. J Exp Psychol Human 33: 1062–1075. 10.1037/0096-1523.33.5.1062 17924807

[14] Kim SY, Kim M-S, Chun MM (2005) Concurrent working memory load can reduce distraction. P Natl Acad of Sci 102: 16524–16529. 10.1073/pnas.0505454102 PMC1283430 16258067

[15] Pratt N, Willoughby A, Swick D (2011) Effects of working memory load on visual selective attention: Behavioral and electrophysiological evidence. Front Hum Neurosci 5. 10.3389/fnhum.2011.00057 PMC3115462 21716633

[16] Baddeley AD (2000) The episodic buffer: A new component of working memory? Trends Cogn Sci 4: 417–423. 11058819 10.1016/s1364-6613(00)01538-2

[17] Jarrold C, Tam H, Baddeley AD, Harvey CE (2011) How does processing affect storage in working memory tasks? Evidence for both domain-general and domain-specific effects. J Exp Psychol Learn 37: 688–705. 10.1037/a0022527 21319919

[18] Oberauer K (2009) Interference between storage and processing in working memory: Feature overwriting, not similarity-based competition. Mem Cognition 37: 346–357. 10.3758/MC.37.3.346 19246349

[19] Baddeley AD (2012) Working memory: Theories, models, and controversies. In: Fiske ST, Schacter DL, Taylor SE, editors. Annu Rev Psychol. pp. 1–29. 10.1146/annurev-psych-120710-100422 21961947

[20] Fan J, Flombaum JI, McCandliss BD, Thomas KM, Posner MI (2003) Cognitive and brain consequences of conflict. NeuroImage 18: 42–57. 12507442 10.1006/nimg.2002.1319

[21] Fan J, Hof PR, Guise KG, Fossella JA, Posner MI (2007) The functional integration of the anterior cingulate cortex during conflict processing. Cereb Cortex 18: 796–805. 17652463 10.1093/cercor/bhm125

[22] Fougnie D, Marois R (2011) What limits working memory capacity? Evidence for modality-specific sources to the simultaneous storage of visual and auditory arrays. J Exp Psychol Learn 37: 1329–1341. 10.1037/a0024834 PMC4156106 21859231

[23] Luck SJ, Vogel EK (1997) The capacity of visual working memory for features and conjunctions. Nature 390: 279–281. 9384378 10.1038/36846

[24] Todd JJ, Marois R (2004) Capacity limit of visual short-term memory in human posterior parietal cortex. Nature 428: 751–754. 15085133 10.1038/nature02466

[25] Vogel EK, Woodman GF, Luck SJ (2001) Storage of features, conjunctions, and objects in visual working memory. J Exp Psychol Human 27: 92–114. 10.1037//0096-1523.27.1.92 11248943

[26] Vogel EK, Machizawa MG (2004) Neural activity predicts individual differences in visual working memory capacity. Nature 428: 748–751. 15085132 10.1038/nature02447

[27] Alvarez GA, Cavanagh P (2004) The capacity of visual short-term memory is set both by visual information load and by number of objects. Psychol Sci 15: 106–111. 14738517 10.1111/j.0963-7214.2004.01502006.x

[28] Olsson H, Poom L (2005) Visual memory needs categories. P Natl Acad of Sci USA 102: 8776–8780. 10.1073/pnas.0500810102 PMC1150822 15937119

[29] Konstantinou N, Lavie N (2013) Dissociable roles of different types of working memory load in visual detection. J Exp Psychol Human 39: 919–924. 10.1037/a0033037 PMC3725889 23713796

[30] Yeh Y-Y, Lin S-H (2013) Two mechanisms of distractor dilution: Visual selection in a continuous flow. Journal of Experimental Psychology: Human Perception and Performance 39: 872–892. 23106375 10.1037/a0030486

[31] Marciano H, Yeshurun Y (2011) The effects of perceptual load in central and peripheral regions of the visual field. Vis Cogn 19: 367–391.

[32] Lavie N, Cox S (1997) On the efficiency of visual selective attention: Efficient visual search leads to inefficient distractor rejection. Psychol Sci 8: 395–398.

[33] Allen RJ, Hitch GJ, Mate J, Baddeley AD (2012) Feature binding and attention in working memory: A resolution of previous contradictory findings. Q J Exp Psychol 65: 2369–2383. 10.1080/17470218.2012.687384 22670689

[34] Fan J, McCandliss BD, Sommer T, Raz A, Posner MI (2002) Testing the efficiency and independence of attentional networks. J Cogn Neurosci 14: 340–347. 11970796 10.1162/089892902317361886

[35] Masson MEJ (2011) A tutorial on a practical Bayesian alternative to null-hypothesis significance testing. Behav Res Methods 43: 679–690. 21302025 10.3758/s13428-010-0049-5

[36] Raftery A (1995) Bayesian Model Selection in Social Research. Sociol Methodol 25: 111–163.

[37] Caparos S, Linnell KJ (2010) The spatial focus of attention is controlled at perceptual and cognitive levels. J Exp Psychol Human 36: 1080–1107. 10.1037/a0020367 20873935

[38] Linnell KJ, Caparos S (2011) Perceptual and cognitive load interact to control the spatial focus of attention. J Exp Psychol Human 37: 1643–1648. 10.1037/a0024669 21767051

[39] Caparos S, Linnell KJ (2009) The interacting effect of load and space on visual selective attention. Vis Cogn 17: 1218–1227.

[40] Baddeley AD, Logie RH (1999) Working memory: The multiple component model. In: Miyake A, Shah P, editors. Models of working memory. New York: Cambridge University Press. pp. 28–61.

[41] Cocchini G, Logie RH, Della Sala S, MacPherson SE, Baddeley AD (2002) Concurrent performance of two memory tasks: Evidence for domain-specific working memory systems. Mem Cognition 30: 1086–1095. 10.3758/bf03194326 12507373

[42] Guerard K, Tremblay S (2008) Revisiting evidence for modularity and functional equivalence across verbal and spatial domains in memory. J Exp Psychol Learn 34: 556–569. 10.1037/0278-7393.34.3.556 18444756

[43] Logie RH, Zucco GM, Baddeley AD (1990) Interference with visual short-term memory. Acta Psychol 75: 55–74. 10.1016/0001-6918(90)90066-o 2260493

[44] Vergauwe E, Barrouillet P, Camos V (2010) Do mental processes share a domain-general resource? Psychol Sci 21: 384–390. 20424075 10.1177/0956797610361340

[45] Barrouillet P, Bernardin S, Portrat S, Vergauwe E, Camos V (2007) Time and cognitive load in working memory. J Exp Psychol Learn 33: 570–585. 10.1037/0278-7393.33.3.570 17470006

[46] Barrouillet P, Bernardin S, Camos V (2004) Time constraints and resource sharing in adults' working memory spans. J Exp Psychol Gen 133: 83–100. 14979753 10.1037/0096-3445.133.1.83

